# Netrin-1 suppresses CCL2-driven macrophage migration: implications for modulating vascular inflammation and aortic sinus enlargement in hyperlipidaemic mice

**DOI:** 10.1016/j.jmccpl.2026.100868

**Published:** 2026-09-01

**Authors:** Vasco Claro, Yanira Riffo-Vasquez, Fulye Argunhan, Albert Ferro

**Affiliations:** a School of Cardiovascular and Metabolic Medicine and Sciences, British Heart Foundation Centre of Research Excellence, King's College London, London, UK; b Institute of Pharmaceutical Science, King's College London, London, UK

**Keywords:** Netrin-1, Atherosclerosis, Monocytes, Macrophages, Inflammation

## Abstract

**Background:**

Atherosclerosis is driven by sustained recruitment and accumulation of monocytes and macrophages within arterial walls through chemokine-mediated pathways. Netrin-1, an immunomodulatory molecule originally identified in neural development, has emerged as a potential regulator of vascular inflammation, though its precise effects on chemokine-driven leukocyte migration remain undefined.

**Methods:**

We investigated netrin-1 effects on macrophage migration *in vitro* using real-time impedance-based assays and assessed systemic effects *in vivo* through intravital microscopy and aortic sinus histology in an inflammation-induced model.

**Results:**

Netrin-1 selectively inhibited CCL2-driven macrophage migration *in vitro*, reducing migration when combined with the chemokine compared to chemokine alone. In an acute inflammation mouse model, systemic netrin-1 pre-treatment showed a trend toward the decrease of monocyte adhesion and transmigration into the tissue while increasing rolling interactions, suggesting impaired firm adhesion and diapedesis. Importantly, hyperlipidaemic LDLR^−^/^−^ mice presented enlarged aortic sinus after being fed a high-fat diet, and this enlargement was not observed when netrin-1 was continuously administered *via* osmotic minipumps.

**Conclusions:**

Our data suggests that systemic netrin-1 selectively inhibits chemokine-driven monocyte migration *in vitro*, with potential implications for reducing vascular inflammatory cell recruitment, an important component of atherogenesis.

## Highlights

• Netrin-1 selectively blocks chemokine-driven macrophage migration
• Systemic netrin-1 administration may reduce leukocyte transmigration and aortic sinus enlargement
• Our results suggest the possibility that netrin-1 may have a therapeutic role in preventing vascular inflammation and atherogenesis

## Graphical abstract

**Figure:**
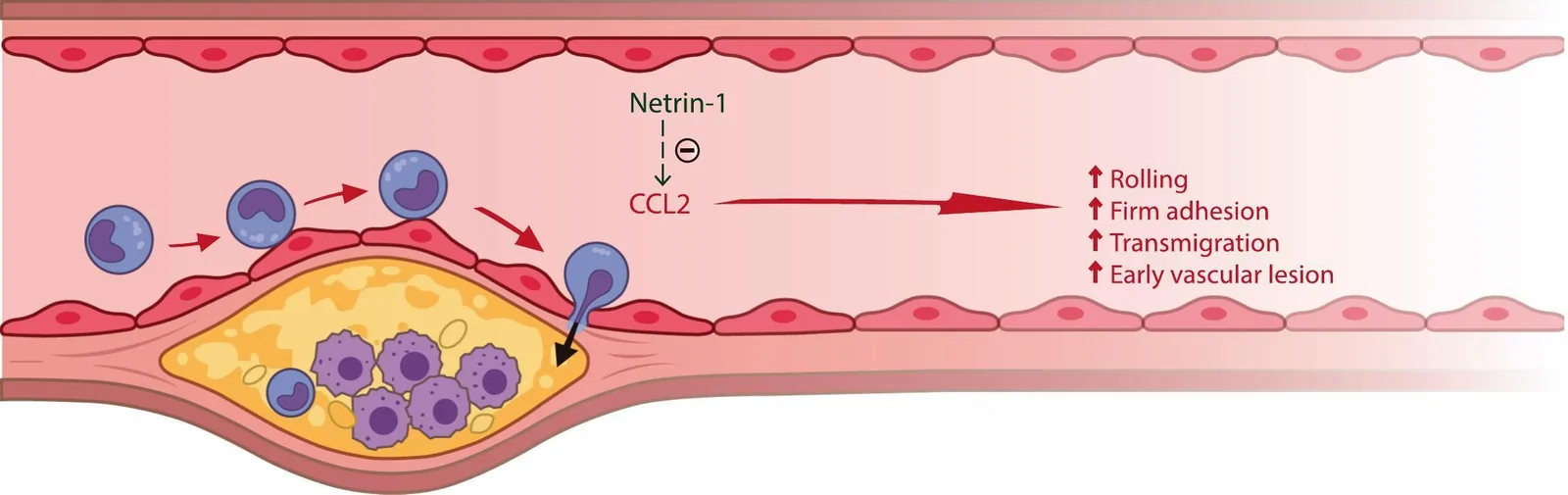

## 1 Background

Atherosclerosis is a chronic inflammatory disease driven by the sustained recruitment and accumulation of monocytes and macrophages within the arterial wall. The early stages of lesion development depend on a tightly regulated sequence of leukocyte trafficking events, including endothelial rolling, firm adhesion, transmigration, and subsequent retention within the tissue [1]. Chemokines such as CCL2 play a central role in this process by promoting monocyte arrest and directional migration across the endothelium, thereby amplifying vascular inflammation and plaque progression [2].

Netrin-1, a laminin-like guidance cue originally identified in neural development, has emerged as an important immunomodulatory molecule in cardiovascular disease. In the context of atherosclerosis, netrin-1 displays seemingly opposing roles depending on its cellular source and localization. Macrophage-derived netrin-1 has been shown to promote macrophage retention within plaques, contributing to disease progression, whereas endothelial-derived or systemic netrin-1 has been shown to play an anti-inflammatory role, with reduced leukocyte recruitment and attenuated inflammation [3]. These contrasting observations highlight the complexity of netrin-1 signalling and underscore the need to better define its functional effects on leukocyte behaviour during inflammation.

At the cellular level, netrin-1 has been reported to influence both leukocyte survival and migration, processes that are critical for determining macrophage burden within atherosclerotic lesions. However, the effects of netrin-1 on the interaction of leukocytes with chemokine-driven migration pathways such as those mediated by CCL2 have not previously been elucidated.

In this study, we investigated netrin-1 effects on macrophage migration *in vitro* and its systemic impact on arterial leukocyte recruitment and early inflammation *in vivo*. Using migration assays, intravital microscopy of monocyte–endothelial interactions, and aortic sinus histology, we show that netrin-1 selectively inhibits chemokine-driven leukocyte migration and this has potential implications in endothelial transmigration.

## 2 Materials and methods

### 2.1 Cell culture and macrophage differentiation

The human monocytic cell line THP-1 (Merck) was maintained in RPMI-1640 (Sigma-Aldrich) medium supplemented with 10% FBS (Gibco) and 1% penicillin–streptomycin (Gibco) at 37 °C, 5% CO₂. To generate macrophages, THP-1 cells were differentiated using 100 ng/mL phorbol 12-myristate 13-acetate (PMA – Sigma-Aldrich) for 24 h, followed by a resting period of 24 h with PMA-free medium to allow full macrophage maturation.

### 2.2 Macrophage migration assays

Macrophage migration was measured using a real-time impedance-based system (xCELLigence™, ACEA) with CIM-plate inserts (Agilent). Lower chambers contained serum-free medium with recombinant human CCL2 (100 ng/mL; Peprotech), recombinant netrin-1 (300 ng/mL; R&D), both, or control medium. After optimisation, 80,000 differentiated macrophages were seeded in upper chambers (Supplementary Fig. 1). Impedance from cells migrating through the membrane was recorded for 21 h as cell index (CI). Migration was quantified as area under the CI–time curve (AUC).

### 2.3 Intravital microscopy of leukocyte–endothelial interactions

All procedures complied with the UK Animals (Scientific Procedures) Act 1986 (Project Licence PP1060882). C57BL/6-Tg(Csf1r-EGFP-NGFR/FKBP1A/TNFRSF6)2Bck/J mice (Charles River), expressing enhanced green fluorescent protein (EGFP) in monocyte/macrophage lineages, were used for real-time fluorescence tracking. Recombinant netrin-1 (500 ng) or vehicle was given subcutaneously 16 h before lipopolysaccharide (LPS; Sigma-Aldrich). Acute inflammation was induced by intrascrotal injection of 6.25 μg LPS, 4 h before surgery.

Intravital microscopy of the cremaster microcirculation was performed under non-recovery anaesthesia (2 mg/kg urethane). After exteriorization of the muscle, tissue was superfused with warm Tyrode's-HEPES buffer and post-capillary venules imaged using a Zeiss Axioskop 2 microscope with a CMOS ORCA-Flash 2.8 camera and 40× water-immersion objective. Under blue laser and dimmed bright field light, monocyte/macrophages and vessel architecture were visualized. At least two 20 s videos per mouse were recorded, each capturing a different vessel within the cremaster muscle (Supplementary Fig. 2). Fluorescent cells in tissue, and those rolling or firmly adherent to endothelium, were counted throughout each recording. Cell counts were averaged across all videos for each mouse and are presented as the mean number of cells per mouse.

### 2.4 In vivo model of diet-induced atherosclerosis

To evaluate long-term systemic netrin-1 effects on vascular pathology, LDLR^−^/^−^ mice (Charles River) were fed a 60% high-fat diet (LBS Biotechnology) throughout the study (Supplementary Table 1, Supplementary Fig. 3). 200 μL recombinant netrin-1 (25 μg/mL) or vehicle was delivered *via* subcutaneous osmotic minipumps (mini-osmotic pump 2006 - Azlet) for 42 days, starting at 12–16 weeks of age. According to the manufacturer instructions, a minipump infusion rate of 0.15 μL/h equates to 3.75 ng/h recombinant netrin-1. Control mice received normal chow. Body weight was monitored. At endpoint, hearts were collected and paraffin-embedded. The aortic roots were sectioned at 8 μm, deparaffinised in xylene and rehydrated through graded ethanol. The sections were stained with Harris haematoxylin and eosin Y. Images were obtained using an Olympus Colourview III camera connected to an Olympus BX51 using CellSens Dimension viewing software. One section encompassing equivalent areas of the aortic sinus was selected from each mouse and analysed in a blinded manner using FIJI ImageJ software. The total aortic sinus area and the area of tissue thickening were quantified using the measurement tools within the software. The areas of tissue thickening measured were those located within the valve leaflets and adjacent to the valve-attachment sites where early signs of atherosclerosis can be detected, as indicated by the black arrows in Fig. 2A–D.

### 2.5 Statistical analysis

Statistical analyses were by one-way ANOVA with Tukey's *post hoc* test for normally distributed data, or Kruskal–Wallis with Dunn's multiple comparisons for nonparametric data. Continuous netrin-1 administration experiments were analysed by one-way ANOVA with Dunnett's *post hoc* test. All tests were two-tailed, with significance set at *P* < 0.05.

## 3 Results and discussion

### 3.1 Netrin-1 inhibits CCL2-driven macrophage migration in vitro

**Fig. 1:**
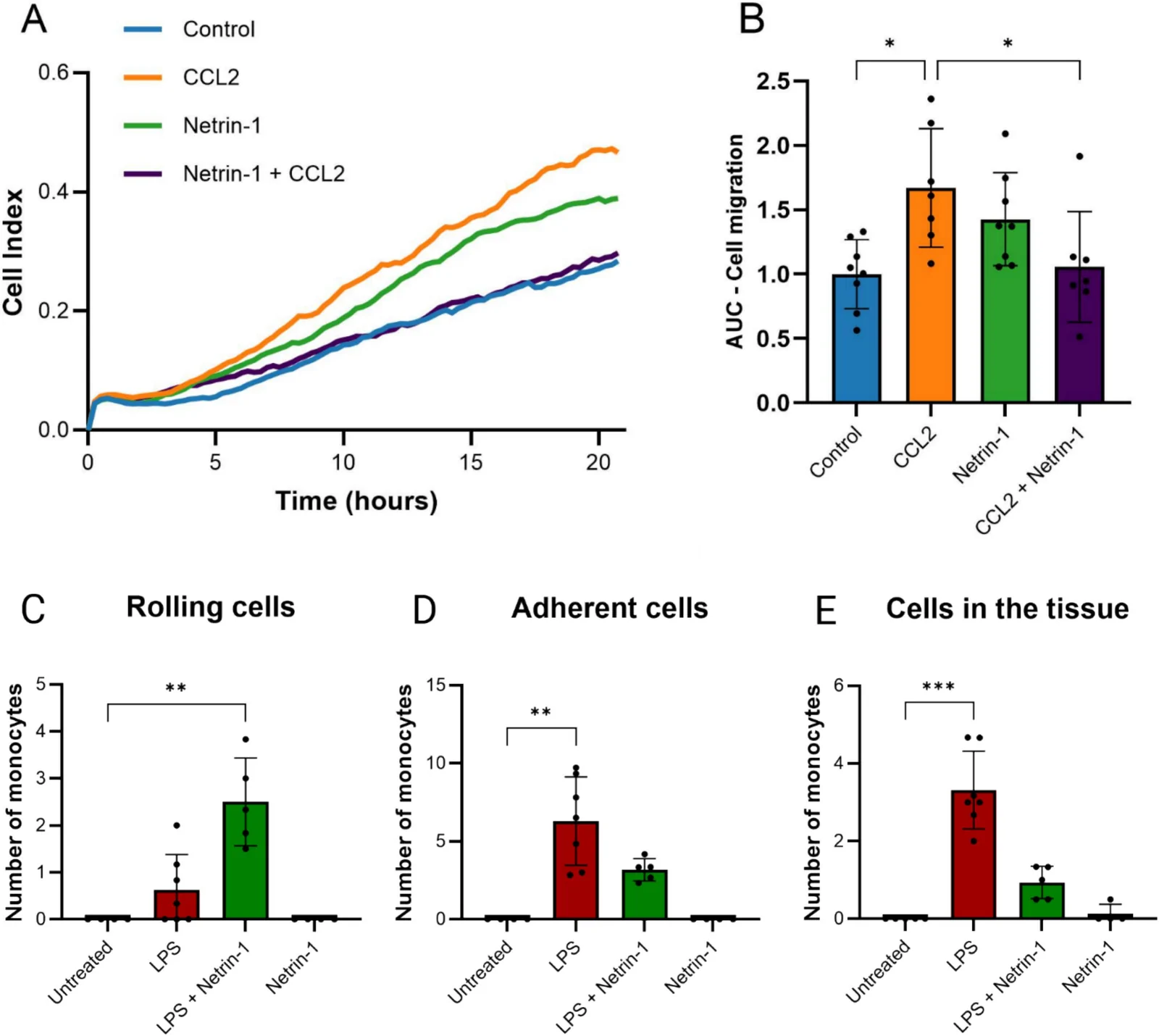
Monocyte/macrophage *in vitro* migration and *in vivo* tissue infiltration following inflammatory stimulation. A–B, Impedance-based migration assay showing typical experiment (A) and accumulated results (B; control and netrin-1 *n* = 8 wells; CCL2 and CCL2 + netrin-1 *n* = 7 wells) examining migration of THP-1–derived macrophages toward chemokine, recombinant netrin-1, or their combination. C–E, Intravital microscopy of the cremaster muscle (4untreated and netrin-1 *n* = 4 mice; LPS *n* = 7 mice and LPS + netrin-1 *n* = 5 mice) showing responses to LPS in terms of mean number of cells detected rolling on the endothelium (C), mean number of cells adherent to the endothelium that remained stationary during 20s recording (D), and mean number of cells detected in the tissue (outside the vasculature) in each mouse (E). Each point but one, from the LPS group, is the mean number of cells counted in two different vessels in the same mouse. Data are presented as mean ± SD. Statistical significance for (B) was determined by one-way ANOVA followed by Tukey's *post hoc* test and for (C-E) using the Kruskal–Wallis test followed by Dunn's multiple-comparison test. *P < 0.05, **P < 0.01, ***P < 0.001 between indicated groups.

We examined whether netrin-1 modulates macrophage migratory responses using a real-time impedance-based migration assay (Fig. 1A and B). CCL2 induced a robust increase in macrophage migration compared with control conditions, confirming its chemoattractant activity. Although netrin-1 alone appeared to elicit a modest, but non-significant, migratory response in macrophages, notably the combination of netrin-1 and CCL2 significantly reduced macrophage migration compared with CCL2 alone, bringing migration levels close to baseline, suggesting that netrin-1 interferes with chemokine-driven directional migration.

### 3.2 Systemic netrin-1 modulates leukocyte-endothelial interactions during acute inflammation in vivo

To determine whether the inhibitory effects of netrin-1 on chemokine-driven migration also occur *in vivo*, we performed intravital microscopy of the cremaster muscle microcirculation following LPS-induced inflammation. EGFP expression is driven by the colony-stimulating factor 1 receptor (Csf1r) promoter in the transgenic mouse model used in this study. As Csf1r is expressed predominantly by monocytes, macrophages, and a subset of dendritic cells, these cells exhibit endogenous EGFP fluorescence and can be visualized by epifluorescence microscopy using 488 nm excitation.

As expected, LPS administration markedly increased monocyte adhesion and transmigration relative to control conditions (Fig. 1D–E). When netrin-1 was administered prior to LPS stimulation, the number of cells adhering to and transmigrating into the tissue showed a trend toward reduction compared to vehicle + LPS treatment, although these differences did not reach significance (*p* = 0.46 and *p* = 0.12 respectively). This was accompanied by an increase in rolling monocytes (Fig. 1C), suggesting that netrin-1 may not block initial endothelial interactions but rather predominantly interferes with the transition from rolling to firm adhesion and subsequent transmigration.

### 3.3 Continuous administration of netrin-1 modulates early aortic sinus enlargement in hyperlipidaemic mice

**Fig. 2:**
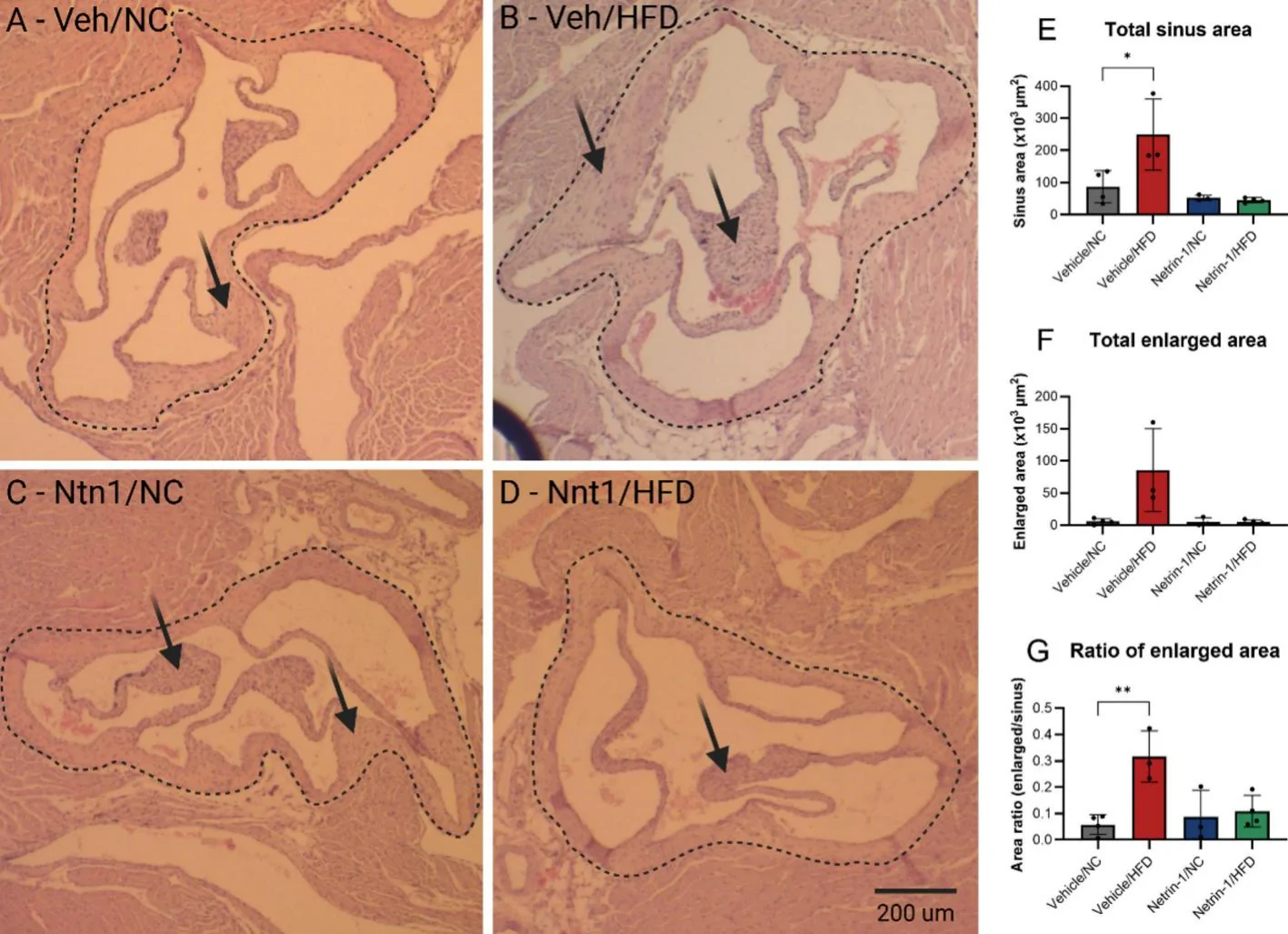
Aortic sinus histology sections and measurement of arterial thickness of wall as cusps from *LDLR*^*−/−*^ mice after being fed with normal chow (NC) or HFD and treated with vehicle or netrin-1. Dotted lines show the sinus outlines. Solid arrows point to thickened aortic cusps indicative of development of plaque. (A – *n* = 4 mice) Vehicle/normal chow, (B – *n* = 3 mice) vehicle/HFD, (C – *n* = 3 mice) netrin-1/normal chow, (D – *n* = 4 mice) netrin-1/HFD. Also shown are accumulated results showing (E) total sinus area, (F) total enlarged area and (G) ratio of enlarged area over to total sinus area. Data are presented as mean ± SD. Statistical significance was assessed using the one-way ANOVA with Dunnett's *post hoc* test*P < 0.05 and **P < 0.01 between groups indicated.

To investigate the longer term effects of netrin-1 on vascular inflammation and pathophysiology, we assessed the effects of prolonged netrin-1 administration on aortic sinus enlargement, an early surrogate marker of atherosclerosis, in fat fed LDLR^−/−^ mice. As expected, LDLR^−/−^ mice fed a HFD developed significant enlargement of the aortic sinus, consistent with early atherosclerotic lesion formation (Fig. 2A–B). Systemic administration of recombinant netrin-1 *via* osmotic minipumps during HFD feeding appeared to attenuate lesion expansion, insofar as the total sinus area in these mice was unchanged from that of mice (both control and netrin-1 treated) fed normal chow, although the difference from those control mice fed HFD did not reach significance (Fig. 2E). The enlarged-area ratio (Fig. 2G) further supports these findings: HFD feeding induced an increase in lesion size relative to normal chow, whereas mice receiving concurrent netrin-1 treatment presented a sinus of similar dimensions to the mice fed with normal chow.

### 3.4 Limitations

Most animals used in our *in vivo* studies were male. For the intravital microscopy experiments, only male mice could be used since the cremaster muscle is only present in males. Also, the experiments involving prolonged netrin-1 administration included only one female mouse in each group except the treatment group. Anatomical, physiological, and biochemical differences between sexes may influence the response to therapeutic interventions, including recombinant netrin-1. Consequently, the effects observed in this study may not fully capture potential sex-related differences in treatment response. Additionally, the sample sizes used in our experiments were relatively small. Future studies should include larger, sex-balanced cohorts to confirm the findings presented here.

## 4 Conclusions

The results of this study suggest that systemic netrin-1 selectively limits vascular inflammatory cell accumulation by disrupting chemokine-driven monocyte/macrophage migration and transmigration, without broadly suppressing leukocyte–endothelial interactions. *In vitro*, netrin-1 minimally affected basal macrophage motility but markedly reduced CCL2-induced migration, consistent with previous reports in RAW264.7 and adipose-tissue macrophages [4], [5]. *In vivo*, intravital microscopy showed a trend toward reduced monocyte adhesion and extravasation together with significantly increased endothelial rolling following LPS stimulation in netrin-1 treated mice compared with controls, suggesting interference with the transition from rolling to firm adhesion and subsequent transmigration. While these observations are consistent with the known suppression by netrin-1 of endothelial adhesion molecule expression, larger samples sizes would be needed to establish a netrin-1–mediated reduction in LPS-induced monocyte recruitment [6], [7], [8].

During vascular inflammation, endothelial netrin-1 is downregulated [9]. Increasing its systemic levels *via* recombinant netrin-1 administration appeared to prevent the aortic sinus enlargement observed in HFD-fed mice, suggesting decreased inflammatory cell recruitment in lesion-prone regions [10], [11], [12], [13]. Human studies similarly show circulating netrin-1 negatively correlates with plaque volume and is lower in coronary artery disease or acute myocardial infarction, supporting a protective role [7], [14].

Overall, netrin-1 is a selective regulator of chemokine-driven monocyte trafficking, potentially influencing early atherosclerotic lesion development. Systemic netrin-1 supplementation may potentially limit vascular inflammation during early atherogenesis.

## CRediT authorship contribution statement

**Vasco Claro:** Writing – original draft, Investigation, Data curation. **Yanira Riffo-Vasquez:** Writing – original draft, Supervision, Methodology. **Fulye Argunhan:** Writing – original draft, Methodology, Investigation. **Albert Ferro:** Writing – review & editing, Writing – original draft, Supervision, Project administration, Funding acquisition.

## Funding

This work was funded by a British Heart Foundation PhD studentship (grant no. FS/18/69/33884).

## Declaration of competing interest

The authors declare no conflicts of interest.
