# Supplementary material for: Netrin-1 suppresses CCL2-driven macrophage migration: implications for modulating vascular inflammation and aortic sinus enlargement in hyperlipidaemic mice
Source: J Mol Cell Cardiol Plus. 2026 Sep 1;18:100868. doi: 10.1016/j.jmccpl.2026.100868 (PMC13570349; doi:10.1016/j.jmccpl.2026.100868)
Supplement: Supplementary file 1 [file mmc1.docx]

**Supplementary material**

**Supplementary figure 1. Optimisation of THP-1-derived macrophage adhesion to E-plates for determining the optimal cell number for the migration assay.** Measurement of the impedance and CI of different cell densities. (A) Representative experiment showing CI as a function of time for the four different cell densities over 21 h. (B) Accumulated results (n = 8). ***P<0.001 and ****P<0.0001 between groups indicated.

**Supplementary figure 2. Intravital microscopy of monocyte–endothelial interactions in the cremaster muscle microcirculation.** Representative intravital microscopy images from mice subjected to vehicle or netrin-1 treatment, with or without LPS stimulation. For each condition, mirrored images are shown: the original recording (left) and the corresponding annotated image (right). The yellow dotted line delineates the endothelial boundary of the vessel. Yellow dotted circles indicate firmly adherent cells that remained stationary during the 20 s recording period. Green dotted circles indicate rolling cells, and the green dotted line traces their trajectory. Red dotted circles denote cells that have transmigrated into the surrounding tissue. (A) Vehicle/vehicle (negative control), representative frame at 10 s. No leukocytes were observed rolling, adherent, or within the tissue during the recording. (B) Netrin-1/vehicle control, representative frame at 10 s. No leukocytes were detected in either the vasculature or tissue. (C) Vehicle/LPS-treated (positive control), representative frames at 0, 10, and 20 s showing rolling, adhesion, and transmigration events. (D) Netrin-1/LPS-treated, representative frames at 0, 10, and 20 s illustrating reduced adhesion and transmigration compared with vehicle/LPS controls.

**Supplementary figure 3. Body weight changes following 6 weeks of normal chow HFD**. Mice fed a 60% high-fat diet for 6 weeks exhibited a significant increase in body weight compared with mice maintained on normal chow. Weight gain was observed in both vehicle- and netrin-1–treated groups receiving HFD. Data are presented as mean ± SD; n = 3–4 per group.

|  | *Negative control group* | *Positive control group* | *Treated control group* | *Treated group* |
| --- | --- | --- | --- | --- |
| *Number* | 4 | 3 | 3 | 4 |
| *%Sex (M/F)* | 75/25 | 67/33 | 67/33 | 100/0 |
| *Age* | 12-16 weeks | | | |
| *Initial Weight (g)* | 28.76 ± 3.63 | 28.45 ± 3.68 | 29.9 ± 3.38 | 26.56 ± 2.23 |
| *Final Weight (g)* | 29.88 ± 2.29 | 33.86 ± 3.01 | 29.58 ± 2.61 | 33.45 ± 2.07 |

**Supplementary table 1. Baseline characteristics and body weight of experimental groups.** Summary of animal numbers, sex distribution, age, and body weight in the negative control (vehicle/vehicle), positive control (vehicle/LPS), treated control (netrin-1/vehicle), and treated (netrin-1/LPS) groups. Mice were 12–16 weeks of age at study initiation. Body weights were recorded at baseline (prior to dietary intervention and/or treatment) and at the experimental endpoint. Values are presented as mean ± SD.
